# Study of nuclear modification factors of deuteron and anti-deuteron in Pb–Pb collisions at $\sqrt{s_{\mathrm{NN}}} =2.76\,\hbox {TeV}$

**DOI:** 10.1038/s41598-022-05584-2

**Published:** 2022-02-02

**Authors:** Feng-Xian Liu, Zhi-Lei She, Hong-Ge Xu, Dai-Mei Zhou, Gang Chen, Ben-Hao Sa

**Affiliations:** 1 School of Mathematics and Physics, Hubei Polytechnic University, Huangshi, 435003 China; 2 School of Mathematics and Physics, China University of Geosciences, Wuhan, 430074 China; 3 Institute of Particle Physics, Central China Normal University, Wuhan, 430079 China; 4 China Institute of Atomic Energy, P.O. Box 275(10), Beijing, 102413 China

**Keywords:** Theoretical nuclear physics, Phenomenology

## Abstract

The nuclear modification factors ($R_{AA}$) of *d* and $\bar{d}$ have been studied using the parton and hadron cascade model plus the dynamically constrained phase space coalescence model in peripheral (40–60%) and central (0–5%) Pb–Pb collisions at $\sqrt{s_{NN}}=2.76\,\hbox {TeV}$ with $|y|<0.5, p_T<20.0 \,\hbox {GeV}/\hbox {c}$. It is found that the $R_{AA}$ of $d, \bar{d}$ is similar to that of hadrons ($\pi ^\pm , p, \bar{p}$) and the $R_{AA}$ of antiparticles is the same as that of particles. The suppression effect of *d* is more significant than that of baryons and mesons in the high-$p_T$ region. The suppression of $R_{AA}$ at high-$p_T$ strongly depends on event centrality and mass of the particles, i.e., the central collision is more suppressed than the peripheral collision. Besides, the yield ratios and double ratios for different particle species, and the coalescence parameter $B_2$ for ($d, \bar{d}$) in *pp* and Pb–Pb collisions are discussed, respectively. It is observed that the yield ratios and double ratios of *d* to *p* and *p* to $\pi$ are similar to those of their anti-particles in three different collision systems, suggesting that the suppressions of matter ($\pi ^{+}, p, d$) and the corresponding antimatter ($\pi ^{-},\bar{p},\bar{d}$) are around the same level.

## Introduction

It is known that quark-gluon plasma(QGP), a new form of nuclear matter characterized by the deconfined state of quarks and gluons, can be produced in heavy-ion collisions at ultra-relativistic energies, such as at the Relativistic Heavy Ion Collider (RHIC) at BNL and Large Hadron Collider (LHC) at CERN. Since a large amount of energy is deposited in the extended QGP matter, it is allowed to create abundant anti-matter ranging from hadrons to light nuclei. Quantitative studies on the production of anti-matter in high energy heavy-ion collisions will shed light on the understanding to the anti-matter to matter asymmetry in our universe. Up to now, numerous experimental results of (anti)hadrons ($\pi ^{-},\bar{p}$, $\overline{\Lambda }$, etc.) and (anti)nuclei ($\overline{d}$, $\overline{^3{He}}$, and $\overline{_{\overline{\Lambda }}^3 H}$, etc.) in *pp*^1–3^ and Pb–Pb^1,2,4–8^ collisions at $\sqrt{s_{\mathrm{NN}}} = 2.76\ \hbox {TeV}$ have been reported.

Transverse momentum spectra of various particle species in nucleus–nucleus (A–A) collisions can be applied to study many important properties of the QGP matter. The microscopic process at low-$p_T$ is dominant by the bulk production. In the intermediate $p_T$ region, the baryon-to-meson ratio shows an enhancement^9–11^, which is the so called “baryon anomaly” not fully understood so far. For the inclusive particle spectra at high-$p_T$, transport properties of the QGP matter can be obtained through jet quenching^12–14^. Experimentally, the nuclear modification factor $R_{AA}$ is usually performed to study the jet quenching effect^1,15–19^.

The $R_{AA}$, which compares the $p_{T}$ distributions of the charged particles in nucleus–nucleus (A–A) collisions to *pp* collisions, is typically expressed as^2^:

1 $$\begin{aligned} R_{AA}(p_T) = \frac{d^{2}N^{AA}_{id}/ d\eta dp_{T}}{\langle T_{AA}\rangle d^{2}\sigma ^{pp}_{id} /d\eta dp_{T}}. \end{aligned}$$

where $N^{AA}_{id}$ and $\sigma ^{pp}_{id}$ denote the charged particles yield per event in A–A collision and the cross section in *pp* collision, respectively. The nuclear overlap function $T_{AA}$ is computed based on the Glauber model^20^.

The study of the $R_{AA}$ plays an important role in understanding the detailed mechanism by which hard partons lose energy traversing the medium^21^. Recent experimental data of $R_{AA}$ in Pb–Pb collision from ALICE^1,2,17,18,22^ and CMS^19^ experiments have been published for a range of charged hadrons. Compared with $R_{AA}$ of hadrons (charged particles, $\pi ,k,p$, etc.), $R_{AA}$ of light (anti)nuclei is not well explained in high energy A–A collision experiments. Therefore we think the properties of $R_{AA}$ of (anti)hadrons and (anti)nuclei in Pb–Pb collisions deserve to be further using theoretical models.

Presently, there are many successful phenomenological models widely used to describe the production of hadrons and light nuclei in relativistic heavy-ion collisions^23,24^, such as the Ultra-relativistic Quantum Molecular Dynamics (UrQMD) approach^25^, a Multiphase Transport (AMPT) model^26^, and the Simulating Many Accelerated Strongly interacting Hadrons (SMASH) approach^27^. For the light (anti)nuclei production in terms of their yields, yield ratios, $p_T$-spectra, flow, etc., either the coalescence models^28–35^ or the statistical thermal approaches^36–39^ are usually employed. The lightest nuclear cluster, i.e., deuterons, has been especially studied to shed light on the nuclei formation process. For example, Ref.^31^ shows the spectra and elliptic flow of deuterons by the IEBE-VISHNU hybrid model with AMPT initial conditions + coalescence model at RHIC and LHC energies. In Ref.^38^ a hydrodynamics + hadronic transport approach is adopted to explore the microscopic evolution process of deuteron production via the $\pi d \leftrightarrow \pi p n$ reaction in Pb–Pb collisions at LHC energy.

In this paper, the production and transverse momentum ($p_{T}$) of final state (anti)hadrons ($\pi ^{+},\pi ^{-}$, $p,\bar{p}$) are simulated by PACIAE model^40^ in *pp* and Pb–Pb collisions at $\sqrt{s_{\mathrm{NN}}}=2.76\ \hbox {TeV}$. And then the dynamically constrained phase-space coalescence (DCPC) model^41^ is applied to deal with the production and properties of light (anti)nuclei ($d,\bar{d}$). Previous results of light (anti)nuclei production for both *pp*^41,42^ and A–A^43–49^ collisions in relativistic energy region, including spectra, energy dependence, scaling property, centrality dependence have been obtained using this framework. In the rest of this paper, we will investigate the properties of nuclear modification factors ($R_{AA}$) of (anti)hadrons and (anti)deuteron in Pb–Pb collisions at $\sqrt{s_{\mathrm{NN}}}=2.76\ \hbox {TeV}$ with the same approach.

The paper is organized as follows: In “Models” section, we concisely introduce the PACIAE and DCPC model. In “Results and discussions” section, our numerical calculation results of the $R_{AA}$ for (anti)hadrons and (anti) deuteron are presented and compared with the available experimental data at LHC. In “Conclusions” section, a brief summary is provided.

## Models

The PACIAE model^40,50,51^ based on PYTHIA 6.4^52^, is designed and expanded to be feasible for lepton-nucleus and nuclear-nucleus (p–p, p–A and A–A) collisions. Compared with PYTHIA , the partonic rescattering process is introduced after the creation of parton initial conditions, while the hadronic rescattering may happen after the hadronization of QCD matter in PACIAE model. In this model, the entire collision process contains four evolution stages as follows:

Firstly, the partonic initial states are created by simplifying nucleus–nucleus collision into numerous nucleon-nucleon (*NN*) collisions according to the collision geometry, Glauber model and *NN* total cross section. Each *NN* collision is described by the PYTHIA model generating quarks and gluons for further evolution. A partonic initial state, also considered as quark-gluon matter (QGM), is reached when all *NN* collisions are exhausted. Secondly, the parton rescattering proceeds via the $2\rightarrow 2$ parton-parton scattering described by the lowest-leading-order perturbative QCD (Lo-pQCD) cross sections^53^. Thirdly, the hadronization process is treated through the Lund string fragmentation approach^52^. Finally, the hadron rescattering is carried out till the exhaustion of hadron-hadron collision pairs or the hadronic freeze-out. One can see^40^ for the detail.

Unlike previous works within PACIAE2.0^40^, here we choose the upgraded version PACIAE2.2^54^ to calculate the nuclear modification factors ($R_{AA}$). In this version, several new physics features such as the final-state transverse momentum anisotropy, a new effective string tension mechanism etc., have been included. Also, an additional chiral magnetic effect(CME) initial charge separation mechanism^55,56^ is introduced. Recently, this approach also has be employed to calculate the “correspondence principle” of $R_{AA}$ between of hadrons and its component quarks in A–A collisions^57^.

PACIAE does not assume equilibrium, such as the other transport (cascade) models UrQMD^25^ and/or AMPT^26^. It just simulates dynamically the whole relativistic heavy-ion collision process from the initial partonic stage to the hadronic final state via the parton evolution, hadronization, and hadron evolution according to copious dynamical ingredients assumptions introduced reasonably. Therefore it is parallel to the experimental nucleus–nucleus collision. These dynamics correctly describe the particle, energy, and entropy developments, etc., while the intensive thermodynamical quantities are not defined in this non-equilibrium regime.

In the theoretical papers, the yield of nuclei usually is calculated in two steps: First, the nucleons are calculated by the transport model. Then, the nuclei are calculated by the phase-space coalescence model based on the Wigner function^58^ or by the statistical model^59^. We proposed a dynamically constrained phase-space coalescence (DCPC) model^41^ to calculate the yield of (anti-)nuclei after the transport model simulations.

From quantum statistical mechanics^60^, one can not precisely define both position $\vec {q}\equiv (x,y,z)$ and momentum $\vec {p} \equiv (p_x,p_y,p_z)$ of a particle in six-dimensional phase space because of the uncertainty principle, $\Delta \vec {q}\Delta \vec {p} \sim h^3$. One can only say this particle lies somewhere within a six-dimensional quantum box or state of volume of $\Delta \vec {q}\Delta \vec {p}$ volume element in the six-dimensional phase space corresponds to a state of the particle. Therefore, one can estimate the yield of a single particle^60^ by

2 $$\begin{aligned} Y_1=\int _{E_{a}\le H\le E_{b}} \frac{d\vec {q}d\vec {p}}{h^3}, \end{aligned}$$

where *H* denotes the Hamiltonian of energy function and $E_a$, $E_b$ are the lower and upper energy threshold, respectively. Analogously, one can compute the yield of the light (anti)nuclei containing N particles with the following integral:

3 $$\begin{aligned} Y_N=\int \cdots\int _{E_{a}\le H\le E_{b}} \frac{d\vec {q}_1d \vec {p}_1...d\vec {q}_Nd\vec {p}_N}{h^{3N}}. \end{aligned}$$

Such as, the yield of a *p*-*n* cluster or deuteron in the DCPC model can be calculated by

4 $$\begin{aligned} Y_{d}=\int \cdots\int \delta _{12}\frac{d\vec {q}_1d\vec {p}_1 d \vec {q}_2d\vec {p}_2}{h^{6}}, \end{aligned}$$

5 $$\begin{aligned} \delta _{12}=\left\{ \begin{array}{ll} 1 \quad \text {if} \quad 1\equiv p, 2\equiv n;\\ \quad m_{d}-\Delta m\le m_{inv}\le m_{d}+\Delta m,\\ \quad q_{12}\le D_{0};\\ 0 \quad \text {otherwise}. \end{array}\right. \end{aligned}$$

where

6 $$\begin{aligned} m_{inv}=\sqrt{(E_1+E_2)^2-(\vec {p}_1+\vec {p}_2)^2}. \end{aligned}$$

Here, the variables $\vec {q}$ and $\vec {p}$ are the coordinates and momentum of the particle in the center-of-mass frame system at the moment after hadronic completion. $m_{d}$ denotes the mass of deuteron, and $\Delta m$ refers to its mass uncertainty; $E_1, E_2$ and $\vec {p}_1, \vec {p}_2$ denote the energies and momenta of the two particles (*p* and *n*); the $q_{12}= |\vec {q}_1-\vec {q}_2|$ is the distance between the two particles. The deuteron is produced by the combination of proton and neutron after the final hadrons have been produced using the PACIAE model.

In Eq. (2), the energy function *H* satisfies $H^2 =(\vec {p}_1 +\vec {p}_2 )^2 + m_{inv}^2$ and the energy threshold satisfies $E_{a,b}^2 = (\vec {p}_1 +\vec {p}_2 )^2 + (m\mp \Delta m)^2$. Thus, the dynamic constraint condition $m-\Delta m\le m_{inv}\le m+\Delta m$ in Eq. (5) is equivalent to $E_{a}\le H\le E_{b}$^61^. Hence we may use the constraint condition $m-\Delta m\le m_{inv}\le m+\Delta m$, instead of $E_{a}\le H\le E_{b}$, to estimate the yield of particle clusters by the phase-space integral.

## Results and discussions

**Figure 1:**
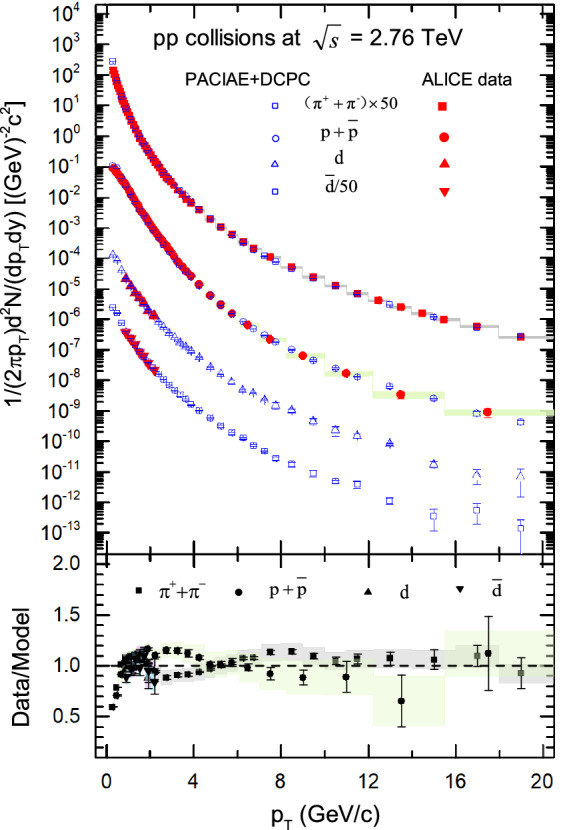
The transverse momentum spectra of charged pions, (anti)protons, and (anti)deuteron computed by PACIAE + DCPC model (the open symbols) in *pp* collisions at $\sqrt{s}=2.76\,\hbox {TeV}$, compared with ALICE results^2,3^ (the solid symbols). The vertical lines (error bars) show the statistical uncertainty and the shaded areas represent the systematic uncertainty of the ALICE results. The spectra have been scaled by the factors listed in the legend for clarity. The lower panels show the deviations of the spectra predicted by our model to ALICE data.

**Figure 2:**
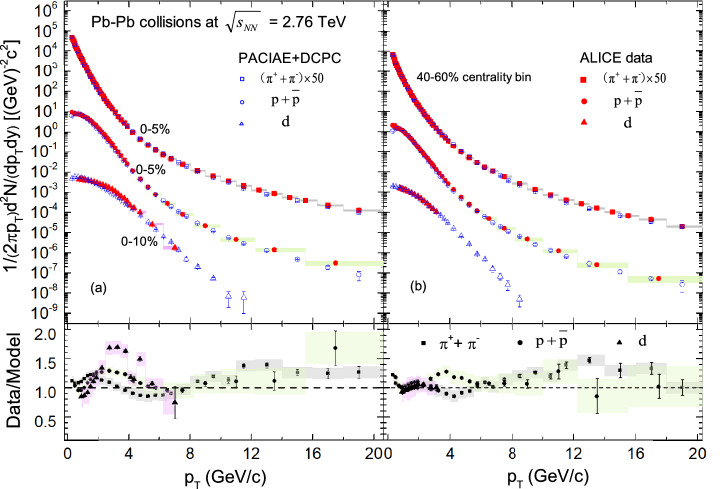
The transverse momentum spectra of charged pions, (anti)protons, and deuteron are presented by PACIAE + DCPC model (the open symbols) in Pb–Pb collisions at $\sqrt{s_{\mathrm{NN}}} = 2.76\,\hbox {TeV}$, compared with ALICE results^2,6,7^ (the solid symbols), (**a**) in centrality bin of 0–5% for $\pi ^++\pi ^-$, $p+\bar{p}$ and 0–10% for *d*, (**b**) in centrality bin of 40–60%, respectively. The vertical lines (error bars) show the statistical uncertainty and the shaded areas represent the systematic uncertainty of the experimental results. The spectra of charged pions have been scaled by the factors 50 for clarity. The lower panels show the deviations of the spectra predicted by our model to ALICE data.

At first, we can obtain the final-state particles in *pp* and Pb–Pb collisions using the PACIAE model^40^. In this simulation, the hadrons are created on the assumption that hyperons heavier than $\Lambda$ are already decayed, and most of model parameters are fixed on the default values given in PYTHIA6.4^52^. We determine the *K* factor, parj(1,2,3) for primary hadrons in PACIAE model by fitting to the ALICE pions and protons transverse momentum spectra data^2^. Here, the *K* factor is introduced to include the higher order and the nonperturbative corrections, parj(1) is the suppression of diquark-antidiquark pair production compared with the quark-antiquark pair production, parj(2) is the suppression of strange quark pair production compared with *u*(*d*) quark pair production, and parj(3) is the extra suppression of strange diquark production compared with the normal suppression of a strange quark. The fitted values of $K=2$ (default value is 1 or 1.5), parj$(1) = 0.15$ (0.1), parj$(2) = 0.50$ (0.3), and parj$(3) =0.60$ (0.4) for *pp* collisions as well as $K=2$, parj$(1) = 0.15$, parj$(2) = 0.38$, and parj$(3) = 0.65$ for Pb–Pb collisions are used in later calculations. Then we generate charged pions and (anti)protons transverse momentum spectra by PACIAE model with $|y|< 0.5$ and $0< p_T < 20\,\hbox {GeV}/\hbox {c}$ at $\sqrt{s_{\mathrm{NN}}}=2.76$ TeV, in *pp* collisions as shown in Fig. 1 and Pb–Pb collisions for centrality bin of 0–5% and 40–60% as shown in Fig. 2, respectively.

Then the yields and transverse momentum spectra of (anti)deuteron were calculated by the dynamically constrained phase-space coalescence model (DCPC) in *pp* and Pb–Pb collisions at $\sqrt{s_{\mathrm{NN}}}=2.76\ \hbox {TeV}$ according to the final hadronic states from the PACIAE model. Here, we choose the model parameter $D_0= 3$ fm and $\Delta m=0.42\, \hbox {MeV}/\hbox {c}$ in *pp* and Pb–Pb collisions^46^. In the end, we can compare the model calculations of the nuclear modification factors for (anti)hadrons and light (anti)nuclei in Pb–Pb collisions at $\sqrt{s_{\mathrm{NN}}}=2.76\ \hbox {TeV}$ to experimental data and study the quenching effect in relativistic heavy-ion collisions.

In Fig. 1, the transverse momentum spectra of charged pions, and (anti)protons computed by PACIAE model (the open symbols) in *pp* collisions at $\sqrt{s}=2.76\ \hbox {TeV}$ within rapidity $|y| < 0.5$ were used to fit model parameters with ALICE results^2^ (the solid symbols). In addition, the transverse momentum spectra of (anti)deuteron calculated by the PACIAE + DCPC model simulation (the open symbols) in *pp* collisions at $\sqrt{s}=2.76\ \hbox {TeV}$ within rapidity $|y| < 0.5$ are also shown in the Fig. 1, which is in agreement with the known ALICE results^3^. The experimental data can be reproduced well at $p_{T} > 4\ \hbox {GeV}/\hbox {c}$, while a certain discrepancy exists between experimental data and model results at $p_{T} < 4\ \hbox {GeV}/\hbox {c}$, especially for low $p_{T}$ pions and intermediate $p_{T}$ protons due to overestimate or underestimate of their spectrum. Therefore, the present model is required for further improvement to a better description of transverse momentum spectra of the final-state hadrons.

Similarly, Fig. 2 shows the transverse momentum spectra of charged pions, and (anti)protons calculated by PACIAE + DCPC model (open symbols) in Pb–Pb collisions at $\sqrt{s_{\mathrm{NN}}} =2.76\,\hbox {TeV}$ for different centrality bins of 0–5% and 40–60% within rapidity $|y| < 0.5$ confronted with ALICE results^2^ (the solid symbols). One can see from Fig. 2 that for $p_T < 3.0\ \hbox {GeV}/\hbox {c}$, the spectra in central collisions becomes harder and there is a mass dependent effect. Both protons and pions transverse momentum spectra are well described by our model in different centrality bins. Then the transverse momentum spectra of deuteron computed by the PACIAE + DCPC model simulation (the open symbols) in Pb–Pb collisions at $\sqrt{s_{\mathrm{NN}}} = 2.76\,\hbox {TeV}$ in both central and peripheral collisions are in agreement with the ALICE data^6,7^, but a large discrepancy of deuterons compared to pions and protons as shown in the lower panel of Fig. 2. This phenomenon can be explained that, according to the deuteron production mechanism, deuteron can be formed by coalescence of ($p + n$). Hence the mass ordering as well as the superposition of the difference of data-to-model ratios for proton and neutron, can lead to the discrepancy of Data/Model ratios for deuterons more larger than that for pions and protons.

**Figure 3:**
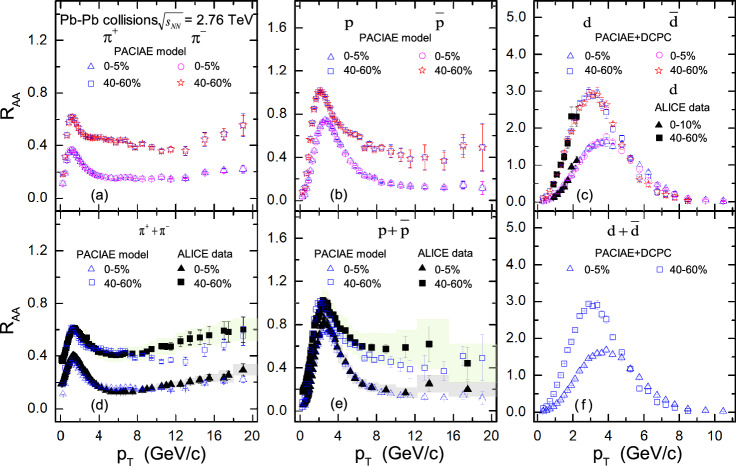
The nuclear modification factor $R_{AA}$ are calculated by PACIAE + DCPC model (the open symbols) for different particle species in 0–5% most central and 40–60% peripheral Pb–Pb collision events at $\sqrt{s_{NN}}=2.76\,\hbox {TeV}$, as a function of $p_{T}$. The ALICE results (the solid markers) for comparison were taken from Ref.^2^ for panel (**d**) and (**e**), and were computed using the data from Ref.^3,6^ for panel (**c**). The vertical lines (error bars) show the statistical uncertainty and the shaded areas represent the systematic uncertainty of the experimental results.

The nuclear modification factor $R_{AA}$ for pion, proton and deuteron is shown in Fig. 3 (the open symbols). Figure 3a–c show the distribution of the nuclear modification factor $R_{AA}$ for the $\pi ^+$, *p*, and *d* compared to their antiparticles $\pi ^-$, $\bar{p}$, and $\bar{d}$, in two different centrality bins. Figure 3d–f show the distribution of $R_{AA}$ versus $p_T$ for combined $\pi ^+ +\pi ^-$, $p+\bar{p}$, and $d+\bar{d}$.

From Fig. 3, one can see that the distribution of the nuclear modification factor $R_{AA}$ for different particle species and different centrality increases with $p_T$ value, reaches a peak, and then decreases with transverse momentum $p_T$, indicating a unified energy loss mechanism is acting on all the different particle species including nuclei at high transverse momentum. And the depression effect of central collision event are more significant than that of peripheral collision, due to a stronger medium modification effect in central collisions.

We notice that the $R_{AA}$ factors of different particles exhibit a maximum for the intermediate $p_{T}$ range, $2.0< p_{T} <4.0\,\hbox {GeV}/\hbox {c}$, a feature generically called the “Cronin effect” (growth of high-transverse momentum cross sections with nuclear size)^62^. For the shapes of the $R_{AA}$, it may be explained that at high-$p_{T}$ the $R_{AA}$ is suppressed owing to the strong partonic energy loss effect. While at low and intermediate $p_{T}$ region, it can be understood by radial boosts and/or the Cronin Effect^63^. For the low-$p_{T}$ region, radial boosts may push particles to higher $p_{T}$ region, leading to a smaller $R_{AA}$. For the enhancement at intermediate $p_{T}$, the Cronin effect due to the multiple nucleon-nucleon scattering effect^64^ tends to transform the longitudinal momentum into the transverse momentum, and finally results in a pronounced peak at the intermediate $p_{T}$ region.

Next, we can see from Fig. 3a–c that the $R_{AA}$ distribution of antihadrons and antinuclei are the same with that of corresponding hadrons and nuclei, showing that the $R_{AA}$ suppression or quenching effect on matter and antimatter is the same in high energy Pb–Pb collisions. It is worth noting, as shown in Fig. 3c,f, that the suppression or quenching effect in the high transverse momentum region is more significant for nuclei than in meson and baryons, this may be interpreted as the nuclei spectra are changed more dramatic due that the so-called “pion wind effect” (protons rescatter with pions gaining higher transverse momentum) induced by hadron rescattering process and baryons-antibaryons $B\overline{B}\rightarrow$ mesons annihilation reactions^38^ influence the transverse momentum spectra of component nucleons for nuclei at high-$p_T$ region.

The solid markers in Fig. 3c–e represent the experimental data^2,3,6^ compared with our simulation results. It is observed that the $R_{AA}$ results of the $\pi ^+ +\pi ^-$, $p+\bar{p}$ and *d* from our simulation are comparable to those of the ALICE data at $p_{T} <10.0\,\hbox {GeV}/\hbox {c}$ within the current errors in Fig. 3c–e; while as $p_{T} > 10.0\,\hbox {GeV}/\hbox {c}$, our simulation is off the data by a small factor due to the particles spectra are slight underestimated at this region. It should be mentioned that the ALICE data $R_{AA}$ of *d* used for comparison in Fig. 3c were calculated according to Eq. (1) based on the experimental data taken from Ref.^3^ for *pp* collisions and Ref.^6^ for Pb–Pb collisions.

**Figure 4:**
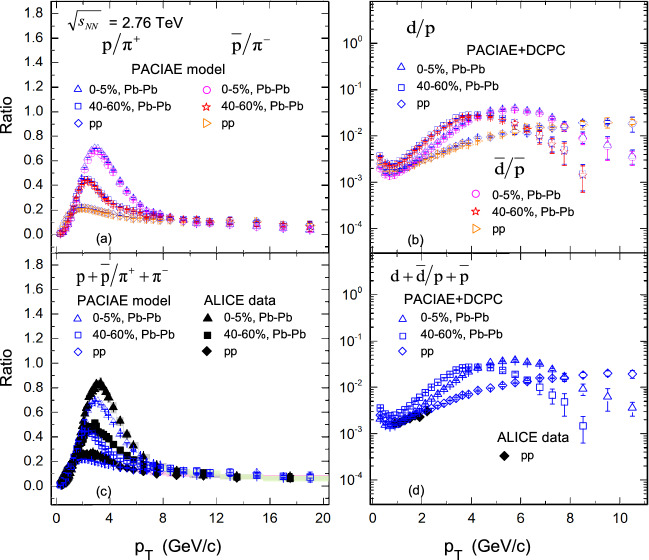
The ratios of (anti)proton to charged-pion and (anti)deuteron to (anti)proton computed by PACIAE + DCPC model (the open symbols) as a function of $p_{T}$ in *pp* collisions, as well as the most central (0–5%) and peripheral (40–60%) Pb–Pb collisions at $\sqrt{s_{NN}}=2.76\,\hbox {TeV}$, respectively. Here, ALICE results (the solid markers) for comparison were take from Ref.^2^ in panel (**c**), and were computed with the data from Ref.^2,3^ in panel (**d**). The vertical lines (error bars) show the statistical uncertainty and the shaded areas represent the systematic uncertainty of the experimental results.

We also perform a particle ratio study versus $p_T$ for (anti)proton to charged pion and (anti)deuteron to (anti)proton in this model. Figure 4a,b, display the ratio distributions of $p/\pi ^{+}$, $\bar{p}/\pi ^{-}$, *d*/*p*, and $\bar{d}/\bar{p}$, respectively. It’s easy to see that the distributions of the ratio for $p/\pi ^{+}, d/p$ are similar to $\bar{p}/\pi ^{-}, \bar{d}/\bar{p}$ in *pp* collisions, central and peripheral Pb–Pb collisions, suggesting a common suppression behavior for the matter and antimatter.

We note that a minimum value of ratios at $p_T = 0.7\ \hbox {GeV}/\hbox {c}$ in Fig. 4b, indicating that deuterons are difficult to produce by protons and neutrons at this region. For the decrease trend below $p_T = 0.7\ \hbox {GeV}/\hbox {c}$, it may be caused by the emitting source volume increases and density of corresponding components decreases^47^. When $p_T > 0.7\ \hbox {GeV}/\hbox {c}$, the ratios increases with $p_T$ value, reaches a peak, and then decreases at higher-$p_T$, which may be a joint result of the dynamics constraints (nuclei with large momentum hard to form) and density of component nucleons ($p{-}n$ correlation hard to occur if density is small).

The ratio distributions of $(p+\bar{p})/(\pi ^{+}+\pi ^{-})$ and $(d+\bar{d})/(p+\bar{p})$ are shown in Fig. 4c,d. It can be seen that for the central and peripheral Pb–Pb collisions, the ratio grows to a maximum value at $p_T \sim 3.0\ \hbox {GeV}/\hbox {c}$ for $(p+\bar{p})/(\pi ^{+}+\pi ^{-})$ and $p_T \sim 5.0\ \hbox {GeV}/\hbox {c}$ for $(d+\bar{d})/(p+\bar{p})$, then decreases as $p_T$ increases. In Fig. 4c,d, the solid markers show the ALICE results^2^ for comparison. Obviously, the $(p+\bar{p})/(\pi ^{+}+\pi ^{-})$ ratio in our simulation shows a similar structure to that in data. The ALICE data $(d+\bar{d})/(p+\bar{p})$ used for comparison in Fig. 4d were computed using data ($p+\bar{p}$) taken from Ref.^2^ and data ($d+\bar{d}$) from Ref.^3^.

To quantify the similarity of the suppression, the double $R^D_{AA}$ ratio were defined, such as the double ratio $R^D_{AA}$ of protons to pions is defined as follows^1^:

7 $$\begin{aligned} R^D_{AA}(p_T) = \frac{R^{(p+\bar{p})}_{AA}(p_T)}{R^{(\pi ^{+} +\pi ^{-})}_{AA}(p_T)}, \end{aligned}$$

where $R^{(\pi ^{+}+\pi ^{-})}_{AA}$ and $R^{(p+\bar{p})}_{AA}$ denote the $R_{AA}$ for the charged pion and proton, respectively. This double ratios constructed using the particle ratios may be properly handled that the dominant correlated systematic uncertainties are between particle species and not between different collision systems.

**Figure 5:**
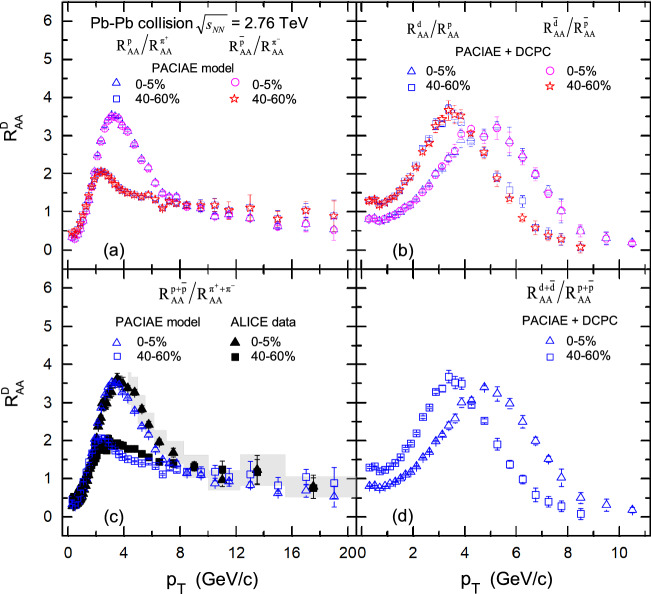
The double ratios $R^D_{AA}$ of (anti)proton to charged-pion and (anti)deuteron to (anti)proton computed by PACIAE + DCPC model (the open symbols) as a function of $p_{T}$ in *pp* collisions, as well as in Pb–Pb collisions of the centrality bins of 0–5% and 40–60% at $\sqrt{s_{NN}}=2.76\,\hbox {TeV}$, respectively. Here, ALICE data (the solid markers) for comparison in panel (**c**), at $p_{T}>4.0\,\hbox {GeV}/\hbox {c}$, were taken directly from Ref.^1^; at $p_{T}<4.0\,\hbox {GeV}/\hbox {c}$, were calculated using the data from Ref^2^. The vertical lines (error bars) show the statistical uncertainty and the shaded areas represent the systematic uncertainty of the experimental results.

Figure 5 shows the double $R^D_{AA}$ ratios of protons ($p, \bar{p}, p+\bar{p}$) to pions ($\pi ^+ ,\pi ^-, \pi ^+ +\pi ^-$) and deuterons ($d, \bar{d}, d+\bar{d}$) to protons ($p, \bar{p}, p+\bar{p}$), as a function of $p_T$, calculated by PACIAE + DCPC in the most central (0–5%) and peripheral (40–60%) Pb–Pb collisions at $\sqrt{s_{NN}}=2.76\,\hbox {TeV}$, respectively. We can see from Fig. 5, that the $R^D_{AA}$ for all particle combinations are generally increasing at low-$p_T$ and decreasing at high-$p_T$. And comparing Fig. 5a,c with Fig. 5b,d, we can also conclude that the suppression effect of the double $R^D_{AA}$ ratio of deuteron to proton is more significant than that of proton to pion, as $p_T > 8\,\hbox {GeV}/\hbox {c}$. For $p_{T} < 8$ GeV/c the double ratios $R^{(p+\bar{p})}_{AA}/ R^{(\pi ^{+} + \pi ^{-})}_{AA}$ in 40–60% peripheral centrality are much lower than that of 0–5% centrality bin. This may reflect a different centrality dependence in magnitude for the two particle species, i.e., for the pions, the suppression becomes more pronounced in the more central collision bins, as expected from the increasingly dense final-state system and longer average path-lengths traversed by hard-scattered partons before fragmenting into final hadrons^19^. However, protons appear to be similar suppressed from peripheral to central events at this $p_{T}$ region.

Besides, it is clear that, as shown in Fig. 5a,b, the distribution of the double $R^D_{AA}$ ratios for *p* to $\pi ^{+}$ and *d* to *p* are the same as that of corresponding antimatter $\bar{p}$ to $\pi ^{-}$ and $\bar{d}$ to $\bar{p}$ , which indicates that matter and corresponding antimatter have the same suppression characteristics. Meanwhile, from Fig. 5c it can be seen that the distribution of the results $R^D_{AA}$ from computed by model simulation are consistent with the ALICE data^1,2^. It should be noted that the experimental values of double ratios $R^{(p+\bar{p})}_{AA}/ R^{(\pi ^{+} + \pi ^{-})}_{AA}$ used for comparison in Fig. 5c, when $p_{T}< 4.0\,\hbox {GeV}/\hbox {c}$, were calculated using data $R_{AA}^{(\pi ^{+} + \pi ^{-})}$ and $R_{AA}^{(p+\bar{p})}$ taken from Ref.^2^, and when $p_{T}>4.0\,\hbox {GeV}/\hbox {c}$, were taken directly from Ref.^1^.

To gain more insight into deuteron production above, we also investigate the coalescence parameter $B_2$ variation based on deuteron and proton data. The coalescence parameter $B_{2}$ plays an important role in depicting the difficulty of (anti-)deuteron production in high energy collisions. And we can obtain the coalescence parameter $B_{2}$ ($p_{T}$) for *pp* and Pb–Pb collisions, from the transverse momentum spectra shown in Figs. 1 and 2, which is inspected as follows^38^:

8 $$\begin{aligned} B_2(p_T) = \frac{\frac{1}{2\pi }\frac{d^3N_d}{p_Tdp_Tdy}|_{p^d_T=2p^p_T}}{\left( \frac{1}{2\pi }\frac{d^3N_p}{p_Tdp_Tdy}\right) ^2}. \end{aligned}$$

**Figure 6:**
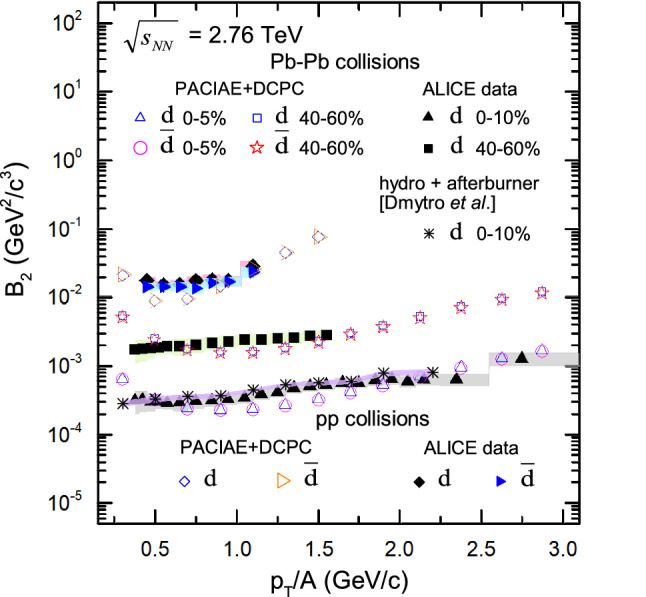
The coalescence parameter $B_{2}$ of (anti-)deuteron, extracted from PACIAE + DCPC model simulation, is compared to ALICE data in *pp*^3^ and Pb–Pb^6,7^ collisions of the most central and peripheral centrality at $\sqrt{s_{\mathrm{NN}}}=2.76\,\hbox {TeV}$, respectively. The vertical lines (error bars) show the statistical uncertainty and the shaded areas represent the systematic uncertainty of the experimental results.

In the Fig. 6, one can see that the distribution of coalescence parameter $B_{2}$ exhibits an increasing trend with the increase of transverse momentum per nucleon ($p_{T}/A$). This increase may be qualitatively explained by position-momentum correlations caused by a radially expanding source^6,7,65^.

To understand this increase better we analyse the coalescence mechanism on deuterons by proton and neutron pairs in our DCPC model, the invariant mass $m_{inv}$ of deuterons can be calculated by Eq. (6) to be $m_{inv}=\sqrt{(E_1 + E_2)^2 - p_{T}^2 - p_{z}^2}$, if we assume that the energy of nucleon $E_1$, $E_2$, and longitudinal momentum $p_{z}$ remain constant, a lower value for $m_{inv}$ will be obtained at the higher-$p_{T}$, leading to the dynamic constraint condition $\Delta m$ in Eq. (3) is much easier to be satisfied. Hence the relative number density probability of p–n pairs enhances at high-$p_{T}$, which ultimately results in the increase of coalescence parameter $B_{2}$ with $p_{T}/A$.

Furthermore, deuteron coalescence parameter $B_{2}$ extracted from the PACIAE + DCPC calculation is quite consistent with the ALICE data^3,6,7^ and the hydrodynamics + hadronic afterburner approach results^38^. One can also see from Fig. 2 that the transverse momentum spectra of proton and deuteron are slightly underestimated, especially for the deuteron in 0–10% Pb–Pb collisions. Hence aiming to get a good $B_{2}$ coalescence parameter, an improved result for the (anti-)proton and (anti-)deuteron spectra should be supplied.

Incidentally, to better understand the nuclear modification factors ($R_{AA}$), especially for the origin of the enhancements at intermediate $p_{T}$ region, we have tested the influence of the effects (such as transverse momentum anisotropy, chiral magnetic effect etc.) and the physics input model parameters (including *K* Factor, parj(1,2,3)) introduced in the PACIAE model, it turns out that these considerations are not enough to explain the origin of the enhancement.

## Conclusions

In the paper, we have studied the transverse momentum spectra of deuteron ($d, \bar{d}$), as well as hadrons ($\pi ^+ +\pi ^-$ and $p+\bar{p}$) at scaled midrapidity $|y| < 0.5$ in *pp* collisions, most central (0–5%) and peripheral (40–60%) Pb–Pb collisions by PACIAE + DCPC model. The key model parameters are determined by fitting pion and proton transverse momentum spectra data. Then, the nuclear modification factors ($R_{AA}$) of charged pions, (anti)protons, and (anti)deuteron, as well as, their yield ratios, double $R^D_{AA}$ ratios and the coalescence parameter $B_2$ with $|y|<0.5$ in peripheral (40–60%) and central (0–5%) Pb–Pb collisions at $\sqrt{s_{NN}}=2.76\,\hbox {TeV}$ have been studied using the PACIAE + DCPC model. It is found that the $R_{AA}$ distribution of light (anti)nuclei ($d, \bar{d}$) is similar to that of hadrons ($\pi ^\pm , p, \bar{p}$), but is more significant for nuclei than meson and baryons at high-$p_T$ region, and the $R_{AA}$ of anti-particles is the same as that of particles. The suppression of $R_{AA}$ at high-$p_T$ strongly depends on event centrality and mass of the particles. Besides, the coalescence parameter $B_{2}$ with transverse momentum per nucleon ($p_{T}/A$) calculated by PACIAE + DCPC model exhibits an increasing trend.

Most of the results predicted by our theoretical model are consistent with existing experimental results, while others are somewhat different, such as the $R_{AA}$ distribution of charged pions at the high-$p_T$. Therefore, the present model is required for further improvement to a better description of transverse momentum spectra of the final-state hadrons and light nuclei. An upcoming meaningful improvement that the production of light nuclei and hypernuclei will be directly implanted into the hadronization stage, can be better employed to study the nuclear modification factors ($R_{AA}$) of nuclei in nuclear collisions.
